# Isolation and Partial Characterisation of Thermophilic Cellulolytic Bacteria from North Malaysian Tropical Mangrove Soil

**DOI:** 10.21315/tlsr2019.30.1.8

**Published:** 2019-01-31

**Authors:** Sandrasekaran Naresh, Balakrishnan Kunasundari, Ahmad Anas Nagoor Gunny, Yi Peng Teoh, Siew Hoong Shuit, Qi Hwa Ng, Peng Yong Hoo

**Affiliations:** Faculty of Engineering Technology, Universiti Malaysia Perlis (UniMAP), P.O Box 77, D/A Pejabat Pos Besar Kangar, 01000, Perlis, Malaysia

**Keywords:** Mangrove, Cellulase, Thermophiles, *Bacillus*, Bakau, Selulase, Termofil, *Bacillus*

## Abstract

This study reports the biodiversity of thermophilic cellulolytic bacterial strains that present in the north Malaysian mangrove ecosystem. Soil samples were collected at the four most northern state of Malaysia (Perak, Pulau Pinang, Kedah and Perlis). The samples obtained were first enriched in nutrient broth at 45°C and 55°C prior culturing in the carboxymethylcellulose (CMC) agar medium. Repeated streaking was performed on the CMC agar to obtain a pure culture of each isolate prior subjecting it to hydrolysis capacity testing. The isolates that showing the cellulolytic zone (halozone) were sent for 16S rRNA sequencing. Total seven isolates (two from Perak, three from Kedah, another two were from Perlis and Penang each) showed halozone. The isolate (KFX-40) from Kedah exhibited highest halozone of 3.42 ± 0.58, meanwhile, the one obtained from Perak (AFZ-0) showed the lowest hydrolysis capacity (2.61 ± 0.10). Based on 16S rRNA sequencing results, 5 isolates (AFY-40, AFZ-0, KFX-40, RFY-20, and PFX-40) were determined to be *Anoxybacillus sp*. The other two isolates were identified as *Bacillus subtilis* (KFY-40) and *Paenibacillus dendritiformis* (KFX-0). Based on growth curve, doubling time of *Anoxybacillus sp*. UniMAP-KB06 was calculated to be 32.3 min. Optimal cellulose hydrolysis temperature and pH of this strain were determined to be 55°C and 6.0 respectively. Addition of Mg^2+^ and Ca^2+^ were found to enhance the cellulase activity while Fe^3+^ acted as an enzyme inhibitor.

## Abstract (ms)

Kajian ini melaporkan biodiversiti strain bakteria selulosa termofilik yang terdapat di ekosistem bakau utara Malaysia. Sampel tanah dikumpulkan dari empat negeri paling utara di Malaysia (Perak, Pulau Pinang, Kedah dan Perlis). Sampel yang diperoleh diperkaya terlebih dahulu dengan brot nutrien pada suhu 45°C dan 55°C sebelum dibiak dalam medium agar karboksimetilselulosa (CMC). Teknik corek digunakan untuk mendapatkan strain tulen setiap bakteria dan bakteria tersebut diuji untuk kapasiti hidrolisis. Strain bakteria yang menunjukkan zon selulosa (*halozone*) telah dihantar untuk penjujukan 16S rRNA. Secara keseluruhan, tujuh strain bakteria (dua dari Perak, tiga dari Kedah, satu dari Pulau Pinang dan satu lagi dari Perlis) menunjukkan zon selulosa. KFX-40 (strain dari Kedah) menunjukkan zon selulosa yang terbesar sebanyak 3.42 ± 0.58, manakala, AFZ-0 (strain dari Perak) menunjukkan zon selulosa yang terkecil sebanyak 2.61 ± 0.10. Hasil penjujukan 16S rRNA menunjukkan bahawa lima (AFY-40, AFZ-0, KFX-40, RFY-20, and PFX-40) daripada tujuh strain yang diperoleh adalah *Anoxybacillus* sp. Sementara itu, dua strain yang lain adalah *Bacillus subtilis* (KFY-40) dan *Paenibacillus dendritiformis* (KFX-0). Analisis keluk pertumbuhan menunjukkan masa ganda dua bagi bakteria *Anoxybacillus* sp. UniMAP-KB06 adalah 32.3 minit. Suhu dan pH optimum untuk strain tersebut adalah 55°C dan 6.0. Penambahan ion Mg^2+^ dan Ca^2+^ meningkatkan aktiviti selulase, manakala, Fe^3+^ membantut aktiviti selulase.

## INTRODUCTION

Mangrove is an ecosystem that protects the land along the seashore from the soil erosion. It is also associated with the defense of shoreline against the flood mainly caused by heavy rainfall and tidal waves. Due to the root system which has a large spread out, the mangrove capable to promote the sedimentation besides claiming land from the sea. Mangrove plays an important role as a natural defense against ecological disasters (Kathiresan 2012). Moreover, mangrove homes variety of marine organisms such as fish, prawns, crabs, amphibians and many more. Mangrove also involved in the purification of water by captivating impurities and heavy metals as well as absorbing the pollutants from the air.

**Figure 1:**
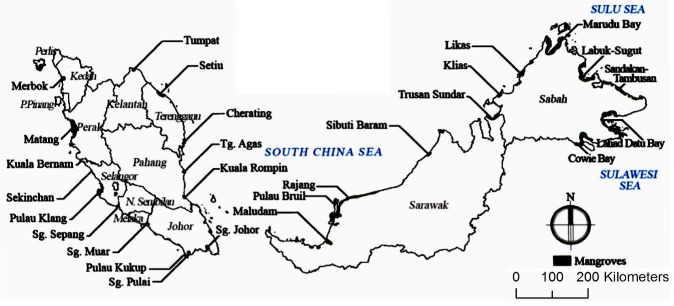
Malaysian mangrove forest distribution (Source: Kanniah *et al*. 2015).

A study carried out by Donato *et al*. (2011) showed that mangroves are the most carbon-rich forests in the tropics. It contains 1,023 Mg carbon per hectare on average, which 49%–98% of carbon is stored in organic-rich soil with the depth ranged from 0.5 m to 3.0 m. Mangrove tree has a very advanced morphological and physiological adaptation. For instance, the pneumatophore is the roots of the mangrove which grows upward to allow the gaseous exchange. Besides that, the mangrove has a cytological pump mechanism which enables them to secrete out excess salts out from their cell (Donato *et al*. 2011). In Malaysia, there are at least 70 mangroves species from 28 families. Out of these 28 families, Rhizophoraceae family is the most dominant in this country. Fig. 1 shows the distribution of the mangrove forest in Malaysia (Kanniah *et al*. 2015).

Organic matters available abundantly in mangrove ecosystem which enables bacteria to bio-mineralise. Leaves and wood of the mangrove plants to the soil are primitively degraded by various microorganisms which contribute to the heterotrophic food chain (Alongi 1994). More than 50% of the organic matters in the mangrove leaves are characterised as water soluble compounds such as tannins and sugars. Nevertheless, the residual of it is made of lignocelluloses which primarily consist of cellulose (Behera *et al*. 2016). Cellulose is a linear chain polymer of D-glucose linked by β-(1,4)-glycosidic bonds and it is the most abundant carbohydrate source in the world.

Cellulose can be broken down using cellulase which is an enzyme. Cellulase can be divided into three types namely endoglucanase (EC 3.2.1.4), exoglucanase (EC 3.2.1.91) and β-glucosidase (EC 3.2.1.21) (Zhang & Zhang 2013). Carboxymethylcellulase is an example of endoglucanase. Cellulase can be produced by fungi, bacteria and plants. Numerous studies were carried out on the degradation of cellulose using fungi due to their capability to produce a large amount of cellulolytic enzyme. Nonetheless, recent studies are more focused on bacteria due to some obvious advantages over the fungal enzyme. Bacteria are considered as potent and functional enzyme producer due to their high growth rate, stability at the harsh condition, and availability of multi-enzyme complexes (Ladeira *et al*. 2015). There are several studies that prove the potential of cellulolytic bacteria isolated from mangroves forest (Behera *et al*. 2014; Soares Júnior *et al*. 2013; Das 2012; Tabao & Monsalud 2010). However, limited studies were available on the isolation of cellulolytic bacteria from Malaysian’ northern states mangrove forests. Thus, in this study, soil samples from the mangrove forests at Perlis, Kedah, Pulau Pinang and Perak were used to isolate cellulose degrading bacteria.

## MATERIALS AND METHODS

### Collection of Mangrove Soil

Soil sample collection was carried out at tropical mangrove ecosystem located in northern states of Malaysia which are Perlis, Kedah, Pulau Pinang and Perak. The locations for the soil collection from these as stated in Table 1. Three random soil collection points were chosen for each location and the soil samples were collected at three different depths for each point. The depths were 0 cm (at the surface), 20 cm and 40 cm. Prior to the sample collection, the temperature of the soil was measured. About 20 g of soil was collected using a sterile spatula and placed into a 50 mL of sterile Falcon tubes. The soil samples were added with 20 mL of sterile distilled water (pH 7.0) and were shaken before measuring the pH and dissolved oxygen content. Then, the Falcon tubes containing soil samples were stored at –20°C until further use (Rastogi *et al*. 2010).

### Culture Enrichment

Ten grams of each soil sample was transferred into a 250 mL Erlenmeyer flask containing 100 mL of sterile distilled water. Subsequently, the flasks were shaken using an incubator shaker (Infors, Switzerland) for 15 min at 150 rpm. For each sample, 1.0 mL of the suspension was pipetted into two different flasks containing 99.0 mL of sterile nutrient broth. The flasks were incubated for two days using incubator shaker at 45°C and 55°C, 150 rpm.

### Screening for Cellulolytic Microorganisms

CMC agar plates were prepared using the composition as stated in Table 2 (Kunasundari *et al*. 2016). After the incubation period, 150 μL of culture from each flask was transferred to the CMC agar plate. A sterile hockey stick was used to spread the culture evenly on the plates. The cultured plates were incubated at their respective temperatures of 45°C (KFX-0 and KFY-40) and 55°C (AFY-40, AFZ-0, KFX-40, RFY-20 and PFX-40) for 24 h. Then, single colonies from the plates were picked and streaked onto the new CMC agar plates. This procedure was repeated a few times until there was no morphological differences observed among the colonies on the same plate.

### Hydrolysis Capacity Testing

Each petri dish was divided into four quadrants. A single colony was picked from the cultured plate and placed in the middle of each quadrant. The plates were then incubated for 24 h at their respective temperatures (45°C or 55°C). After incubation, the plates were flooded with Gram’s iodine solution (2 g potassium iodide and 1 g iodine in 300 mL water) for 5 min (Ferbiyanto *et al*. 2015; Bradner *et al*. 1999). The cellulolytic index for each culture was calculated using the following Equation 1 (Bradner *et al*. 1999):

(1) $$\text{Celluloytic Index}=\frac{\left(\text{Diameter of clear zone}-\text{Diameter of bacterial colony}\right)}{\text{Diameter of bacterial colony}}$$

### Strain Identification and Phylogenetic Tree Construction

Morphological identification was carried out by observing the colony appearance on the CMC agar plates. The 16S rRNA gene sequence analysis was done by Macrogen, Inc. Korea. The data obtained from 16S rRNA sequencing were analysed by a similarity search in the GenBank database using the BLAST-N programme at National Center Biotechnology Information (http://www.ncbi.nlm.nih.gov). The gene sequences were aligned and the phylogenetic tree was established using a neighbour-joining method at 1,000X bootstraps with MEGA 7.0 software (Gusakov *et al*. 2011).

The primers 27F 5′ (AGA GTT TGA TCM TGG CTC AG) 3′ and 1492R 5′ (TAC GGY TAC CTT GTT ACG ACT T) 3′ were used for the PCR. The PCR reaction was performed with 20 ng of genomic DNA as the template in a 30 μL reaction mixture by using a EF-Taq (SolGent, Korea) as follows: Activation of Taq polymerase at 95°C for 2 min, 35 cycles of denaturation at 95°C for 1 min, annealing at 55°C, and extension at 72°C for 1 min. Final extension was done for 10 min at 72°C. The amplification products were purified with a multiscreen filter plate (Millipore Corp., Bedford, MA, USA). Sequencing reaction was performed using a PRISM BigDye Terminator v3.1 Cycle sequencing Kit. The DNA samples containing the extension products were added to Hi-Di formamide (Applied Biosystems, Foster City, CA). The mixture was incubated at 95°C for 5 min, followed by 5 min on ice and then analysed by ABI Prism 3730XL DNA analyzer (Applied Biosystems, Foster City, CA).

### Growth Profile Analysis

Growth profile was established using the strain that showed the highest cellulolytic capacity (KFX-40). Two loopful of pure culture was transferred into 100 mL of sterile nutrient broth in a 250 mL Erlenmeyer flask and shaken at 55°C, 150 RPM using an incubator shaker (Infors, Switzerland). A control containing 100 mL nutrient broth in a 250 mL Erlenmeyer flask was also prepared. Sampling was carried with an interval of 1 h. The cell concentration was obtained by measuring the optical density (OD_600nm_) using a UV-Vis Spectrophotometer (UV-1800, Shimadzu). The measurements were taken until three consecutive readings were observed to decrease. A growth curve of cell concentration against time was plotted. The growth kinetics was analysed using Polymath software to determine the specific growth rate and doubling time.

### Enzyme Production and Recovery

Two loopful of *Anoxybacillus sp*. UniMAP-KB06 were transferred from CMC agar plate into a 250 mL flask containing 100 mL of nutrient broth, pH 7.0. The flask was incubated at 55°C, 150 RPM using the incubator shaker until the culture reached its log phase (6–8 hours). Subsequently, 3% of the culture (OD_600nm_ ~ 0.9) was transferred to the 97 mL of cellulase production broth and incubated for 12 h at 55°C, 150 RPM (Kunasundari *et al*. 2017). Production broth was modified with the addition of 0.1 g/L of CaCl_2_.2H_2_O (Table 2) as described by Liang *et al*. (2010). Then, the cultures were transferred into 50 mL centrifuge tubes and centrifuged using Gyrogen centrifuge at room temperature and 4,000 RPM for 20 min. The cells pellets were discarded whereas the supernatant was used as the crude enzyme.

### Enzyme Assay

Five hundred microliter of crude enzyme was added to 500 μL of 2.0% CMC (substrate) prepared in 0.05 M citrate buffer at pH 4.8 and incubated at 50°C in a water bath for 30 min. Subsequently, 3.0 mL of DNS reagent was added to stop the reaction. The solution was boiled for another 5 min for colour development. Next, it was cooled using an ice bath for 2 min to stabilise the colour. Two hundred microliter of sample was diluted with 2.5 mL of distilled water and subjected to absorbance determination at 540 nm against a blank containing all the reagents excluding the enzyme. The absorbance reading was used to determine the cellulase activity by determining the glucose concentration based on the glucose standard curve (Ghose 1987).

### Enzyme Activity and Stability

#### Effect of temperature on enzyme activity and stability

The enzyme stability under different incubation temperatures (40°C, 50°C, 60°C, 70°C, 80°C, and 90°C) was determined using standard assay condition (2% CMC in 0.05 M citrate buffer with pH 4.8) (Ghose 1987). The stability study was carried out for 3 h and 0.5 mL of sample was withdrawn at an interval of 1 hour to test for the residual enzyme activity.

#### Effect of pH on enzyme activity and stability

The cellulase activities at different pH were experimented using citrate (4.8, 5.0 and 6.0) and Tris-HCl (7.0, 8.0, and 9.0) buffers under standard assay conditions (Ghose 1987). Stability study was performed by incubating the enzyme mixed with respective buffer solution at 50°C for 3 h. With every hour interval, 0.5 mL was withdrawn to measure the residual enzyme activity (Ghose 1987).

#### Effect of metal ions on enzyme activity and stability

The effects of metal ions on enzyme activity were studied by dissolving the respective salts (FeSO_4_.7H_2_O, CaCl_2_.2H_2_O, and MgSO_4_.7H_2_O) separately in 0.05 M citrate buffer pH 4.8 under standard assay conditions (Ghose 1987). The metal ion-citrate buffer solution was mixed with enzyme and incubated at 50°C for 3 h. To test for residual enzyme activity, 0.5 mL of sample was withdrawn at an interval of 1 h (Ghose 1987).

## RESULTS AND DISCUSSIONS

### Collection of Mangrove Soil

**Figure 2:**
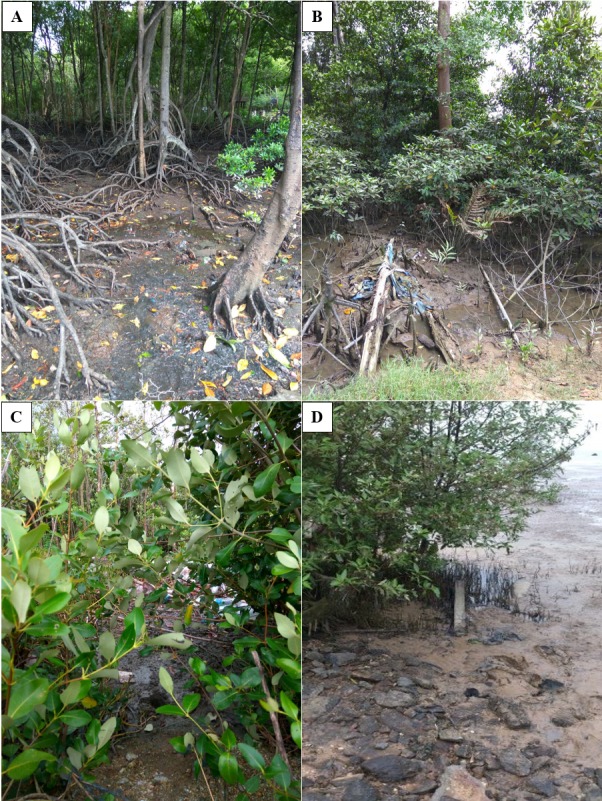
(A), (B), (C) and (D) shows the mangrove soils where the sampling were done.

Soil samples were collected from the mangrove swamp. The information on the temperature, dissolved oxygen content, and pH of the collected soil samples at various mangrove ecosystems from northern states of Malaysia are shown in Table 3. Fig. 2 shows the surroundings of mangrove ecosystems where the soil samples were obtained for this research. The pH in the range of 6.2–6.8 was recorded from all the sampling locations. It can be deduced that the mangrove ecosystems in northern states of Malaysia are slightly acidic. Unlike the popular belief that the mangrove water to be alkaline due to the dissolved calcium of the shells and corals, the presence of sulphur reducing bacteria causes the water to be slightly acidic to neutral (Gusakov *et al*. 2011).

The highest temperature recorded was 36.8°C at Kuala Trong, Perak. Meanwhile, the lowest temperature was 27.1°C at Taman Paya Bakau, Perak. However, these readings are also influenced by the weather and temperature during the sampling day (Table 1). Besides pH and temperature, the dissolved oxygen was measured during sampling. The dissolved oxygen was found to be in the range of 15.7%–19.6%. The dissolved oxygen content in soil gave some rough idea whether the microorganisms surrounding the specific ecosystem are mainly aerobes or anaerobes. These readings revealed that the mangrove soils among the sampling locations did not show much variation in terms of temperature, pH and dissolved oxygen.

### Enrichment and Screening of the Cellulolytic Bacterial Strain

**Figure 3:**
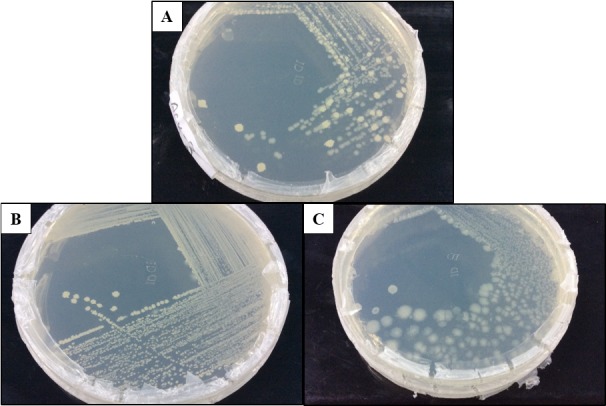
(A) Initial screening for cellulolytic microorganisms, (B) and (C) are pure isolates.

The enrichment of the soil sample was carried out to enhance the growth of all the microorganisms present in the ecosystem. This is important to enable the non-dominant microorganisms to have equal survival chances before using selective media. Subsequently, the culture was grown on the CMC agar plates to screen for the microorganisms that producing cellulase. Fig. 3 (A) shows the streak plate that was obtained from the culture on spread plate using the soil sample from Kedah (KFX-40). As two distinct colonies were observed, further subculturing was performed by streaking a single colony of different morphology appearance. Repeated streaking was performed to obtain pure culture as in Figs. 3 (B) and (C). The same method was applied to all the samples. A total of 12 isolates were obtained based on the morphological characterisation. Four cultures were isolated from Perak (AFX-0, AFY-40, AFZ-0, and ASY-20), four from Kedah (KFX-0, KFX- 40, KFY-40, and KFZ-20), two from Penang (PFX-0 and PFX-40), and two from Perlis (RFY-20 and RFY-40). The pure cultures obtained were tested for hydrolysis capacity to evaluate the cellulolytic properties.

### Hydrolysis Capacity Testing

Table 4 and Table 5 show the cellulolytic capacity of each isolate after staining with Gram’s iodine at 45°C and 55°C, respectively. It can be observed that the Isolate KFY-40 has the highest cellulolytic index of 3.40 ± 0.01 for the temperature of 45°C and the lowest was KFX-0 with the cellulolytic index of 3.21 ± 0.58 in the same temperature group. Meanwhile for 55°C, isolate KFX-40 has the highest cellulolytic index of 3.42 ± 0.58 and the lowest was by isolate AFZ-0 (2.61 ± 0.10). The larger the hydrolysis capacity means the higher the production of the cellulase enzyme by the culture. Hence, isolate AFX-40 was selected as the potential cellulase producing strain at 55°C. Similar findings were documented by Hatami and Alikhani (2008) where the cellulolytic bacteria was detected based on halozone formation.

### Strain Identification

Only 7 out of 12 cultures have shown good hydrolysis capacity testing which were then subjected to genotypic identification. The 16S rRNA sequences obtained from Macrogen, Inc. were analysed using BLAST-N approach. Table 6 shows the identified isolates by 16S rRNA sequencing with respective designated names. An isolate was assigned to the corresponding species based on the 99% identity with 16S rRNA sequences deposited in GenBank. It was determined that all the isolates were from Bacillaceae family. Bacillaceae is a member of the phylum Firmicutes, gram-positive, rod- or coccus-shaped bacteria which produces endospore. Most of the isolates belong to the relatively new genus *Anoxybacillus*, which emerged in the year 2000. *Anoxybacillus* spp. can be either aerobes or facultative anaerobes. These bacteria are moderately thermophile and exhibited ability to withstand high alkaline conditions. Hence, *Anoxybacillus* spp. strains have been proposed as suitable candidates for numerous bioprocessing applications (Goh *et al*. 2013). Various lignocellulosic enzymes such as xylanases and cellulases were found to be secreted by the members of the bacterial genera of *Bacillus* and *Anoxybacillus*, and possess high temperature tolerance. For instance, a study documented that the extracellular xylanase activity *Anoxybacillus flavithermus* TWXYL3 showed thermostability up to 85°C, meanwhile, the optimum temperature was found to be 65°C (Ellis & Magnuson 2012).

*Bacillus subtilis* is an aerobic bacterium and capable to produce spores in extreme conditions for survival. There were a few studies carried out on *B. subtilis* for cellulase production (Ma *et al*. 2015; Andriani & Park 2006; Mawadza *et al*. 2000) which indicated the potentiality of the bacterium. *B. subtilis* was said to be the best studied Gram-positive bacterium and model organism for various other studies. The genus *Paenibacillus* comprises facultative anaerobic, endospore-forming bacteria (Ash *et al*. 1993). *Paenibacillus dendritiformis* is known for its capability as a phosphate solubilising bacterium. It is used in the production of biofertiliser which is safer compared to the chemically synthesised fertilizer which emits hydrogen fluoride gas. It can form endospore just like *Anoxybacillus* sp. and *B. subtilis* in order to thrive the harsh conditions (Grady *et al*. 2016). *Paenibacillus terrae* ME27-1 was isolated by Liang *et al*. (2014) which showed enzyme activity (CMCase) of 2.08 U/mL at optimal pH of 5.5 and optimal temperature of 50°C.

### Phylogenetic Tree Construction

**Figure 4:**
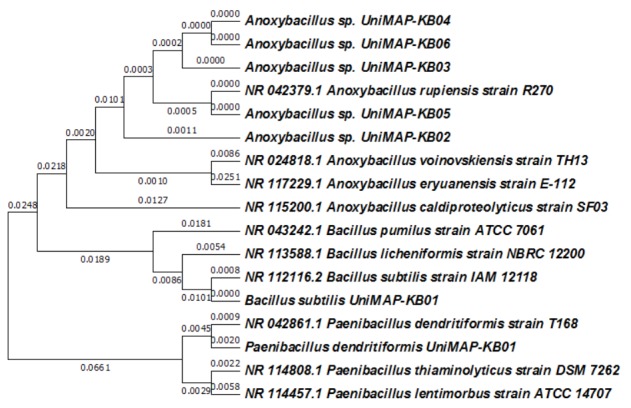
Evolutionary relationships of taxa generated using MEGA 7.0 software.

Neighbour-Joining method was employed to construct the phylogenetic tree for the identified isolates. The Maximum Composite Likelihood method was employed to compute the evolutionary distances and expressed in the units of the number of base substitutions per site. From the phylogenetic tree (Fig. 4), it can be observed that the isolate *Anoxybacillus* sp. UniMAP-KB03, KB04, and KB06 are very closely related to each other. Comparatively, it can be seen based on the phylogenetic tree that all the *Anoxybacillus* sp. isolated in this study are similar to the *Anoxybacillus rupiensis* strain R270. *A. rupiensis* strain R270 is a large rod-shaped gram-positive bacterium which is highly motile. This endospore forming bacterium is an obligate thermophile which grows between 35°C to 67°C with an optimal growth temperature of 55°C. It can grow in a wide range of pH (5.5–8.5). The optimal growth pH is in a slightly acidic range (6.0–6.5) (Derekova *et al*. 2007). However, there is a slight variation in the growth temperature of the isolated strain *Anoxybacillus* sp. UniMAP-KB06. This particular strain does not grow at the temperature of 35°C. Besides, to our knowledge, there are limited studies reported on the cellulolytic activity of *A. rupiensis* (Ghaffari *et al*. 2011). Thus, the stability of cellulase produced by this strain was tested. On the other hand, the *Bacillus subtilis* UniMAP-KB01 and *Paenibacillus dendritiformis* UniMAP-KB01 were found to be distinct when compared to the existing strains in NCBI database.

### Growth Profile Analysis

**Figure 5:**
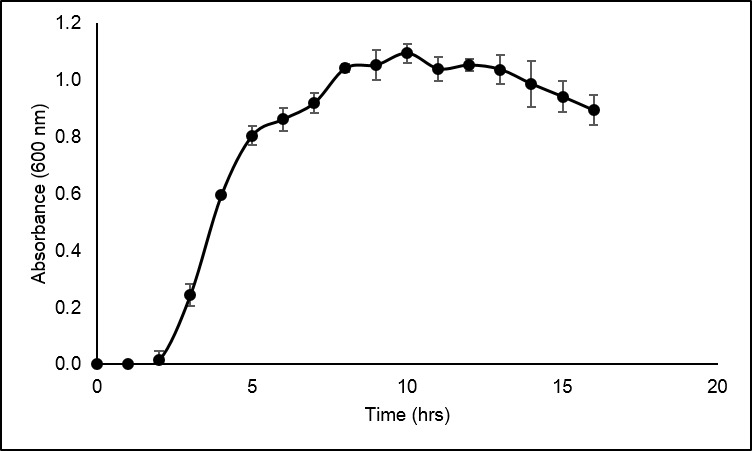
Growth curve of *Anoxybacillus* sp. UniMAP-KB06.

Fig. 5 shows the growth profile of *Anoxybacillus* sp. UniMAP-KB06 in nutrient broth. The exponential phase lasted for 6 h which started at 2nd and ended at the 8th hour. The determination of the exponential phase is crucial to ensure the cells are active prior testing for cellulolytic activities. Thus, the inoculum used throughout this study was prepared by growing it in the nutrient broth for 6 to 8 h with the optical density in the range of 0.8–0.9. Equation 1 was used to calculate the specific growth rate prior determining the doubling time.

(1) $$X_{t}=X_{n}\;{\text{exp}}^{mt}$$

where,

*X_t_* = cell concentration at particular time
*X*_0_ = initial cell concentration
*m* = specific growth rate
*t* = time

**Figure 6:**
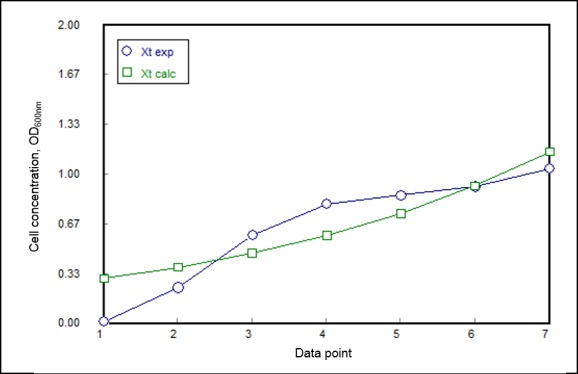
Growth kinetic plot using POLYMATH software.

The initial cell concentration was 0.017. Subsequently, the specific growth rate of this bacterium was determined to be 1.29 hr^−1^. A regression plot (Fig. 6) was established to analyse the data using POLYMATH software. *X_t_* calc indicates the concentration of biomass at the specific time calculated based on the model equation (Equation 1). Meanwhile, the *X_t_* exp represents the experimental values obtained for the cell concentrations. The plot was verified by using the linear regression (R^2^) value which was computed to be 0.9113. This suggests only 8.87% of the total variation could not be explained by the model. Besides that, the adjusted R^2^ value was determined to be 0.8936 which is very near to experimental R^2^. This indicate a high correlation between adjusted and experimental values. Meanwhile, root mean square deviation (RMSD) and variance were calculated to be 0.0395 and 0.0153, respectively. These small values of RMSD and variance denoted the error for the data obtained is insignificant. The doubling time of the bacterium was determined to be 32.3 min using the following Equation 2. It can be deduced that the *Anoxybacillus* sp. UniMAP-KB06 has a short doubling time indicates the potential of this strain to yield a larger amount of biomass in a shorter time period. This property can be exploited to produce cellulase in a shorter duration.

(2) $$t_{d}=\frac{\text{ln}2}{m}$$

where,

*t_d_* = doubling time
ln = log base e
*m* = specific growth rate

### Enzyme Activity and Stability at Different Temperatures

**Figure 7:**
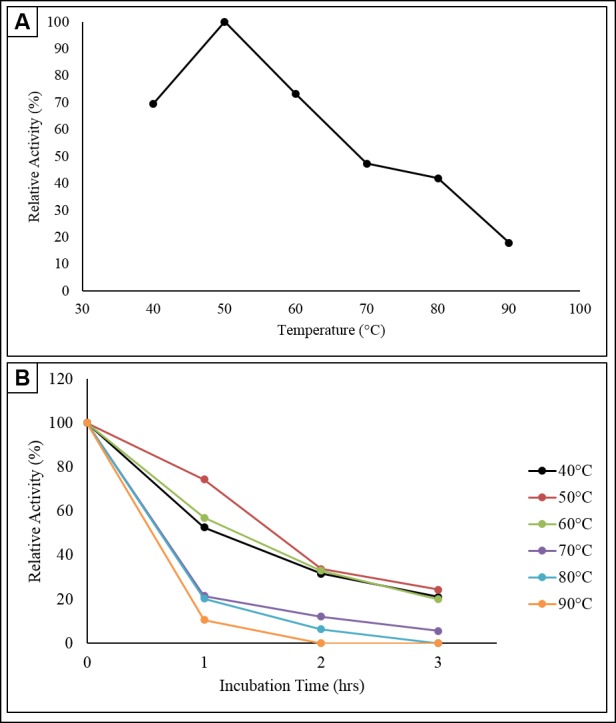
(A) Effect of temperature on enzyme activity and (B) effect of temperature on enzyme stability.

The sample at 50°C was fixed as a control as it was the standard condition for cellulase assay. The relative enzyme activity was appointed as 100% to function as a basis for calculating the cellulolytic activity of other samples. It was observed from Fig. 7(A) that there was an increase in relative enzyme activity about 30.43% when the temperature raised from 40°C to 50°C. The highest relative activity was obtained at 50°C, implying that is the optimum temperature for this strain. The relative enzyme activity at 40°C and 60°C were 69.57% and 73.37% suggesting the temperature required for cellulose hydrolysis by this strain is comparatively higher than mesophile-secreted enzymes. There was a negative slope in enzyme activity beyond 50°C. At 90°C, the residual relative enzyme activity was only 17.93%. This obeys the theory that the enzyme-catalysed reaction will only rose to a certain limited temperature which was known as optimum temperature, and decrease above the temperature as denaturation occurred. Similar findings reported by Lee *et al*. (2008) on cellulase produced by *B. amyoliquefaciens* DL-3 which also showed an optimum enzyme activity at 50°C.

For the stability test, the initial enzyme activity at 0 h for every temperature was appointed as 100%, to act as a basis for relative enzyme activity evaluation during the 3 h incubation period. A declining trend was observed as the period of incubation increased in all the temperatures ranging from 40°C–90°C (Fig. 7(B)). This indicates that the cellulase synthesised by *Anoxybacillus sp*. UniMAPKB06 could not able to withstand high temperature for a prolonged duration. The enzyme relative activity for 40°C shows a tremendous drop of 47.37% after 1 h of incubation. Yet, the relative activity for 50°C only decreased by 25.58% after 1 h of the incubation period. This gives evidence that the enzyme produced was more stable at their optimum temperature. Besides, for the stability test at 90°C, the relative activity has significantly reduced up to 89.36% for the first hour and no enzyme activity was detected for subsequent sampling. This indicated that the enzymes had denatured and were not stable at 90°C. After 3 h of incubation, the relative activities were 21.05% (40°C), 24.42% (50°C), 20.00% (60°C), 5.61% (70°C) while 0.00% for 80°C and 90°C. These depict cellulase produced by *Anoxybacillus* sp. UniMAP-KB06 is moderately thermostable.

### Enzyme Activity and Stability of Crude Enzyme at Different pH

**Figure 8:**
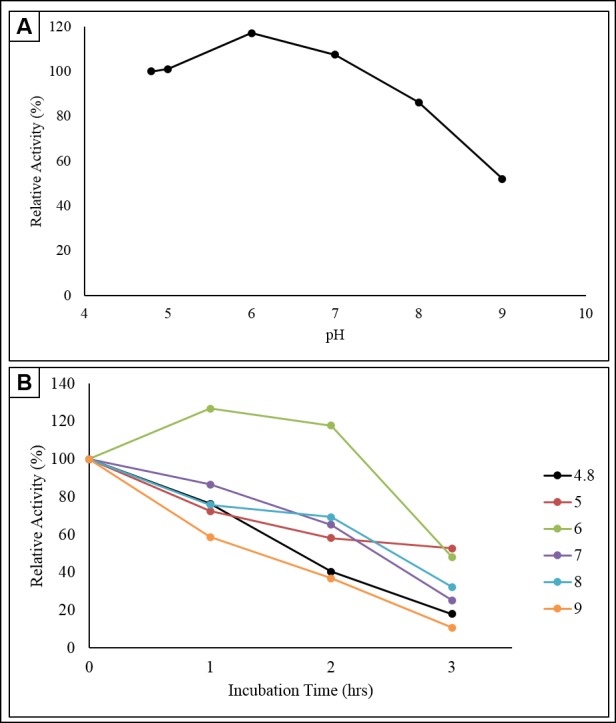
(A) Effect of pH on enzyme activity, and (B) effect of pH on enzyme stability.

Test at pH 4.8 was selected as a control where the relative enzyme activity was appointed as 100% to serve as a basis for calculating the remaining relative enzyme activity at different pH. The results from Fig. 8(A) illustrates that the enzyme activities at pH 5.0, 6.0 and 7.0 were higher than the control. The cellulase secreted by *Anoxybacillus* sp UniMAP-KB06 can withstand a broader range of pH when comparing with standard assay conditions. There were reduction in enzyme activity at pH 8.0 (49.85 ± 1.85 mU/mL) and 9.0 (30.16 ± 2.13 mU/mL) when compared to the control (57.85 ± 2.82 mU/mL). Variation of pH of the medium could change the ionic form of the active site, which led to the decreased enzyme affinity towards the substrate. Besides, alteration in pH usually causes the enzyme to lose its three-dimensional structure. This isolate showed the highest relative activity of 117.02% at pH 6.0 indicating that is the optimal condition for the cellulase produced.

The results are similar to the finding made by Ariffin *et al*. (2006) where endoglucanase synthesised by *B. pumilus* EB3 demonstrated the highest activity at pH 6.0. Besides, the study carried out by Ellis and Magnuson (2012) on the xylanase synthesised by *A. flavithermus* TWXYL3 states that the enzymes possessed a bimodal pH optimum, with maximal activity at pH 6.0 and pH 8.0. As a whole, more than 50% of relative enzymes activity was demonstrated from the range of pH 4.8 to pH 9.0. Therefore, the enzyme synthesised by *Anoxybacillus* sp. UniMAP-KB06 was considered functional at a broad range of pH conditions.

For stability study, the initial enzyme activity at 0 h for every tested pH was appointed to be 100%, functioned as a basis for the relative enzyme activity evaluation for the 3 h incubation period. Figure 8(B) revealed that this enzyme functioned at its best at pH 6.0 as it able to retain the activity for a minimum of 2 h incubation. Increase in relative enzyme activity from 0 to first hour incubation by 26.79% (from 34.46 ± 2.82 mU/mL to 43.70 ± 2.82 mU/mL) was detected. The CMCase activity still remained high (40.62 ± 1.8 mU/mL) at the 2nd hour where the relative enzyme activity is 17.86%, higher than the 0 h. The stability of the enzyme synthesised by *Anoxybacillus sp*. UniMAP-KB06 drop drastically after the 2nd hour of incubation for pH 6.0. At end of the third hour, the relative enzyme activity recorded was 17.98% with enzyme activity of 16.62 ± 1.85 mU/mL.

Based on the graph plotted, it can be deduced that the enzymes were stable at the range of pH 6.0–8.0 for the duration of 2 h as the relative enzyme activity was more than 50% for the entire period of incubation. The result is in agreement with research done by Lin *et al*. (2015) where the cellulase produced by *B. subtilis* YJ1 were stable at pH 6.0–7.5. The stability test for different pH is essential to determine the ability of cellulase for being utilised for industrial application. For instance, Wang *et al*. (2010) reported that new xylanase thermoalkaline *Anoxybacillus sp*. E2 expressed in *E. coli* BL21 (DE3) could reach 90% of maximal enzyme activity at pH 6.6–8.6 while maintaining more than 80% of catalytic property at pH 4.6–12.0. These properties make the newly obtained xylanase as a suitable candidate in the pulp and paper industries, especially for the paper deinking process.

### Enzyme Activity and Stability at Different Concentrations of Various Metal Ions

The control used was the enzyme that incubated at standard condition of temperature 50°C, pH 4.8. The enzyme activity for control was set at 100% which serves as a basis for calculating the remaining relative enzyme activity. Different metal ions provide different effects on the reaction catalysed by enzymes. Calcium chloride (CaCl_2_.2H_2_O), magnesium sulphate (MgSO_4_.7H_2_O) and iron sulphate (FeSO_4_.7H_2_O) at 1 mM and 5 mM were added to CMC substrate medium separately.

**Figure 9:**
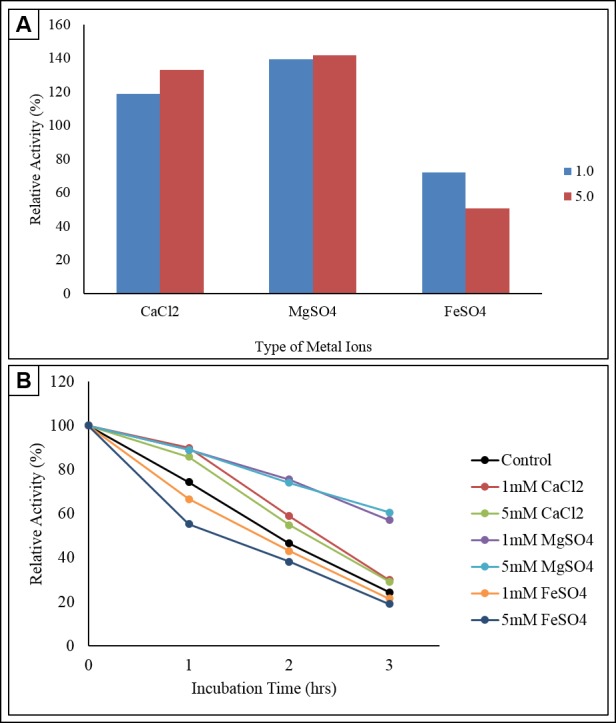
(A) Effect of metal ions on enzyme activity and (B) effect of metal ions on enzyme stability.

From Fig. 9(A), the relative enzyme activity was increased by 18.99% and 32.91% in the presence of 1 mM and 5 mM of CaCl_2_.2H_2_O compare to the control. The highest enzymatic activity of 141.77% was achieved using 5mM MgSO_4_.7H_2_O. Therefore, it can be deduced that the presence of 5 mM of Mg^2+^ promotes the enzyme activity to function optimally in the medium. The relative enzyme activity in 1 mM of Mg^2+^ was slightly lower (139.24%; 67.70 ± 2.13 mU/mL). The increase in Mg^2+^ ions concentration from 1 mM to 5 mM only enhanced the enzyme reaction by 2.53%. Trace concentration of metal ions are believed to improve the substrate binding affinity of the enzyme while providing conformation stability in the catalytic site.

Lin *et al*. (2015) and Zeng *et al*. (2016) found out that the metal cations at 1 mM and 5 mM act as enhancer or activator of cellulase produced by *B. subtilis* YJ1 and *T. virens*. Another study recorded that metal ions such as Na^+^ (sodium ion), K^+^ (potassium ion), Ca^2+^ (Calcium ion), Mg^2+^ (Magnesium ion) and Mn^2+^ (Manganese ion) improved the cellulase activity produced by *B. amyoliquefaciens* DL-3. Besides, the presence of Ca^2+^ for *Clostridium thermocellum*, D-endoglucanase is reported to stabilise the enzyme against thermal denaturation and increase substrate binding affinity (Champasri *et al*. 2015). The results documented by Zeng *et al*. (2016) also showed that Ca^2+^ is an activator which can significantly promote cellulase activity.

Of the three metal ions tested, FeSO_4_.7H_2_O repressed the enzyme activity by 27.85% and 49.37% at the concentrations of at 1 mM and 5 mM, respectively. This indicates that Fe^3+^ ion act as an inhibitor of the crude cellulase of this isolate. This statement can be reinforced with the study documented by Tejirian and Xu (2010) on inhibition of cellulase-catalysed lignocellulosic hydrolysis by iron and two other oxidative metal ions and complexes.

The initial enzyme activity at 0 h for every tested metal ion with respected concentration was appointed to be 100%, functioned as a basis to evaluate the relative enzyme activity for 3 h incubation period. As illustrated in Fig. 9(B), the enzyme activity remained stable in CaCl_2_.2H_2_O which were 55.39 ± 1.85 mU/mL and 36.31 ± 2.82 mU/mL at concentration of 1 mM, 59.70 ± 2.82 mU/mL and 38.16 ± 2.82 mU/mL for concentration of 5 mM at the first 2 h of incubation period. The enzyme activity decreased during the 3rd hour to 18.46 ± 1.85 mU/mL (1 mM) and 20.31 ± 1.85 mU/mL (5 mM). This indicates that the presence of Ca^2+^ ion could maintain the enzyme activity for certain duration. Relative activity of 30.00% and 29.20% remained after 3 h of incubation for both 1 mM and 5 mM concentration of CaCl_2_.2H_2_O.

The relative enzyme activity for MgSO_4_.7H_2_O for the first 2 h incubation was maintained high with respect to 1 mM and 5 mM concentration and reduced slightly to 57.14% and 60.63% at the end of the 3rd hour. Medium supplemented with Mg^2+^ was determined to retain the relative activity of cellulase produced by *Anoxybacillus sp*. UniMAP-KB06 up to 50% while FeSO_4_.7H_2_O was observed to be an inhibitor. The initial CMCase activity in 1 mM and 5 mM of FeSO_4_.7H_2_O recorded were 31.39 ± 1.85 mU/mL and 28.93 ± 2.13 mU/mL, which dropped drastically to 66.67% and 55.32% following 1 h of incubation respectively. For both concentrations, the relative activity remained at the end of experiments were very only 21.57% and 19.15%.

Gaur and Tiwari (2015) documented that the organic-solvent-thermostable alkalophilic cellulase excreted from *Bacillus vallismortis* RG-07 showed higher residual activity in the presence of 5 mM Ca^2+^ (125.4%), Mg^2+^ (115.6%) and Fe^3+^ (100.8%). It is essential to investigate the stability of cellulase with metal ions as production medium usually contain traces of CaCl_2_.2H_2_O, MgSO_4_.7H_2_O, and FeSO_4_.7H_2_O. As metal possess complex action towards enzyme activity, determination of optimum concentration is crucial for better medium preparation and storage stability as they act as a prominent factor for commercialisation of enzyme (Sayem *et al*. 2006).

## CONCLUSION

Soil samples were collected from five different locations in four northern states of Malaysia to isolate thermophilic cellulose degrading bacteria. Seven isolates were screened and subjected to 16S rRNA sequencing. Five isolates were found belong to the genus of *Anoxybacillus*, while the others were *Bacillus subtilis*, and *Paenibacillus dendritiformis*. *Anoxybacillus* sp. strain UniMAP-KB06 exhibited highest hydrolysis capacity. Analysis of growth profile revealed that the doubling time was 32.3 min. Activity and stability studies showed that the enzyme produced by the *Anoxybacillus* sp. UniMAP-KB06 attained maximum activity and stability at temperature of 50°C and pH 6.0. The effect of metal ions study indicated that Mg^2+^ and Ca^2+^ promote the cellulase activity while Fe^3+^ acted as an inhibitor.

## Figures and Tables

**Table 1:** Soil sampling locations and conditions.

State	Location	Latitude	Longitude	Weather	Surrounding temperature
Perlis	Kuala Perlis	N6°23′28.5″	E100°07′59.5″	Light rain	23°C–28°C
Kedah	Kuala Kedah	N6°06′17.6″	E100°17′04.8″	Mostly cloudy	29°C–32°C
Pulau Pinang	Balik Pulau	N5°20′21.5″	E100°11′50.0″	Passing clouds	24°C–25°C
Perak	Kuala Trong	N4°42′45.1″	E100°41′12.8″	Light rain	30°C–31°C
	Taman Paya Bakau, Lumut	N4°12′40.9″	E100°38′48.6″	Overcast	25°C–30°C

**Table 2:** Composition of basal medium (Kunasundari *et al*. 2016).

No.	Compounds	Amount (g/L)
1	KH_2_PO_4_	1.36 g
2	MgSO_4_.7H_2_O	0.20 g
3	NaCl	2.00 g
4	(NH_4_)_2_SO_4_	1.00 g
5	FeSO_4_.7H_2_O	0.01 g
6	CMC	3.00 g
7	Yeast Extract	1.00 g
8	Agar Powder	15.00 g

**Table 3:** Summary of soil sample analysis data.

Locations	Sampling point label	Temperature (°C)	Dissolved oxygen (%)	pH
Taman Paya Bakau, Lumut, Perak	AFX	27.1–29.1	18.7–19.5	6.6–6.8
	AFY	29.9–30.1	17.8–19.2	6.3–6.6
	AFZ	29.8–30.9	18.9–19.3	6.2–6.7
Kuala Trong, Terong, Perak	ASX	31.6–36.8	16.9–19.6	6.2–6.7
	ASY	32.0–32.7	15.7–19.4	6.7–6.8
	ASZ	31.6–31.9	18.0–18.7	6.5–6.7
Balik Pulau, Pulau Pinang	PFX	35.8–36.3	16.2–19.3	6.5–6.7
	PFY	34.6–36.1	17.8–18.9	6.7–6.8
	PFZ	35.4–36.1	17.6–18.6	6.7–6.8
Kuala Perlis, Perlis	RFX	35.1–36.2	17.3–18.6	6.5–6.6
	RFY	34.9–36.1	16.9–17.2	6.6–6.8
	RFZ	35.3–36.1	18.6–19.1	6.6–6.7
Kuala Kedah, Kedah	KFX	34.6–36.1	16.9–19.2	6.2–6.4
	KFY	31.2–33.9	17.5–19.4	6.4–6.9
	KFZ	31.6–32.3	17.8–18.4	6.5–6.7

*Note*: For each sampling points, the range of the temperature, pH, and dissolved oxygen were reported for three different depths of soil (0, 20, and 40 cm).

**Table 4:** Cellulolytics of isolated strains grown at 45°C.

Isolates	Cellulolytics index*	Figures
KFX-0	3.21 ± 0.58	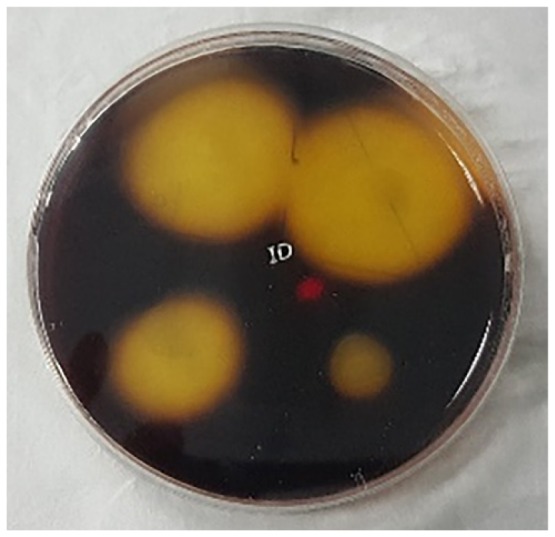
KFY-40	3.40 ± 0.00	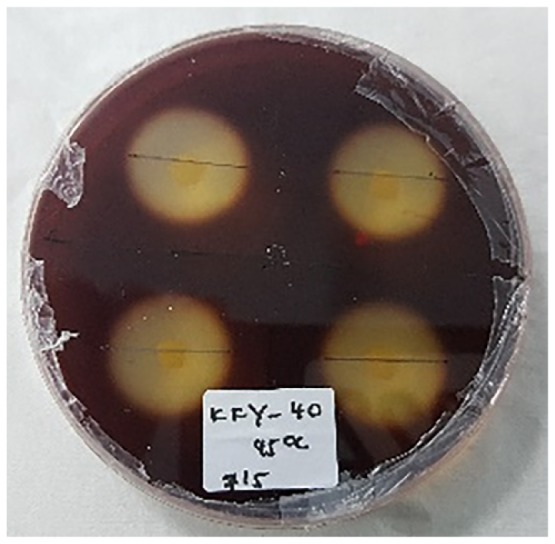

Note:

* Calculated based on the average of quadruplicate.

**Table 5:** Cellulolytic indexes of isolated strains grown at 55°C.

Isolates	Cellulolytic index*	Figures
AFY-40	3.02 ± 0.31	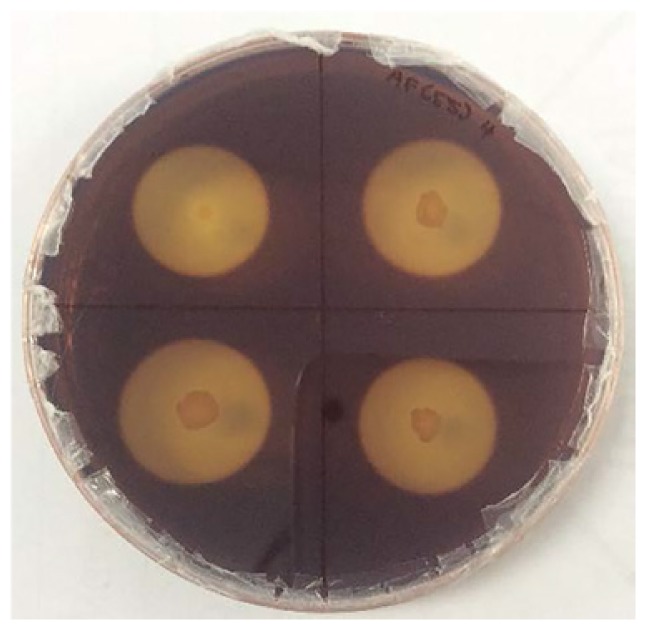
AFZ-0	2.61 ± 0.10	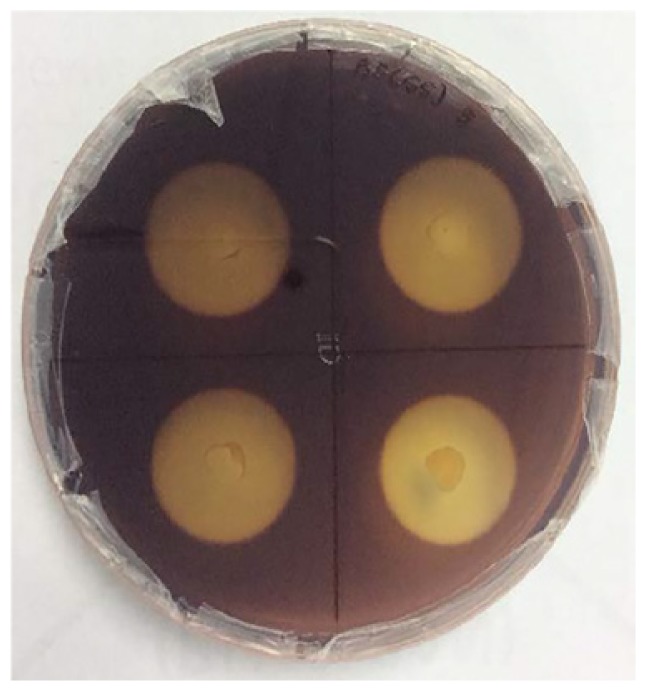
KFX-40	3.42 ± 0.58	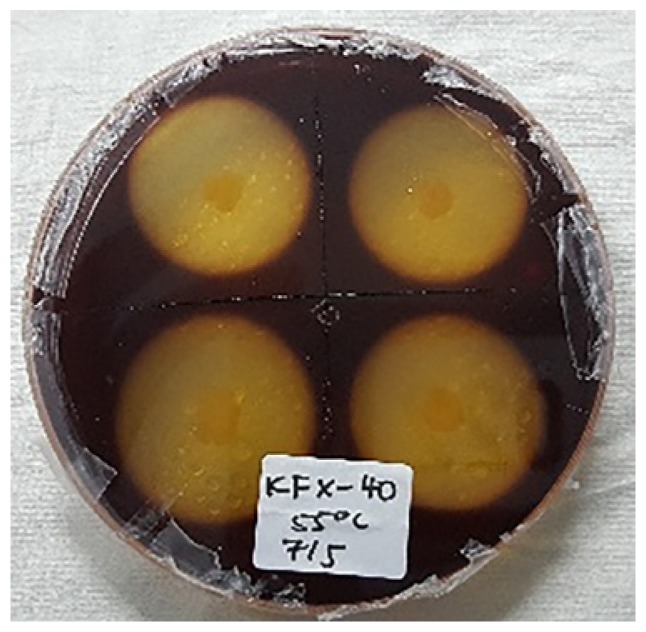
RFY-20	3.06 ± 0.53	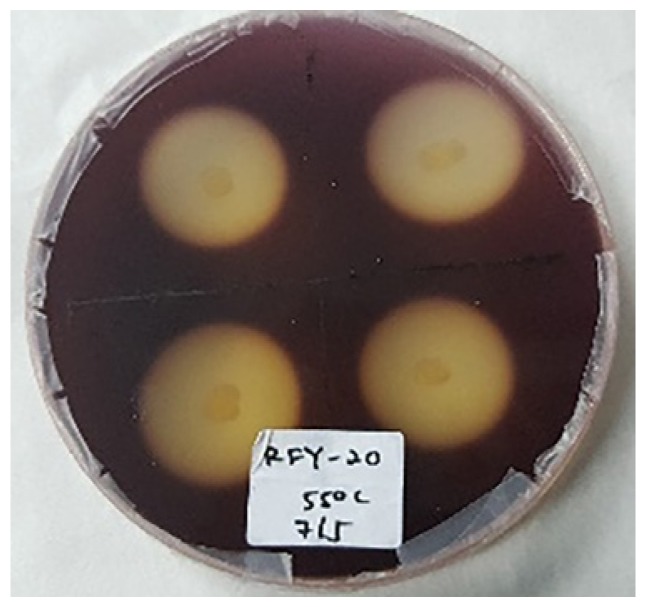
PFX-40	3.21 ± 0.10	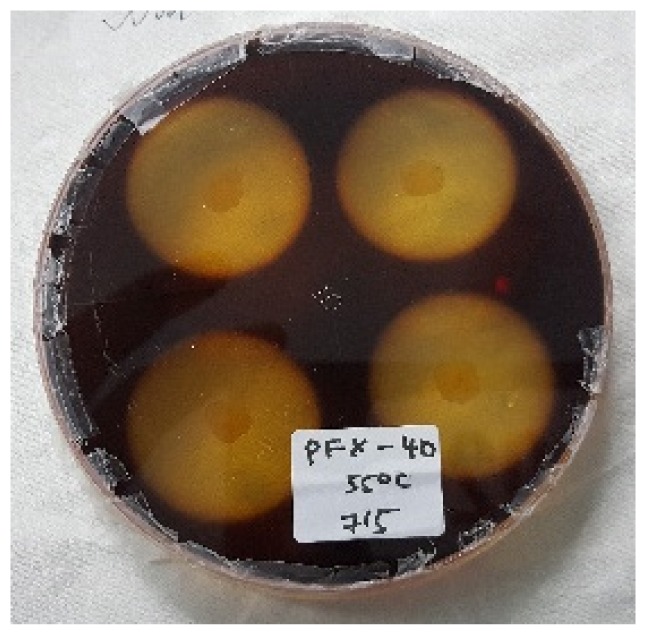

Note:

* Calculated based on the average of quadruplicate.

**Table 6:** Identified isolates by 16S rRNA sequencing with respective designated names.

Isolates	Species name	Designation
AFY-40	*Anoxybacillus* sp.	UniMAP-KB04
AFZ-0	*Anoxybacillus* sp.	UniMAP-KB05
KFX-0	*Paenibacillus dendritiformis*	UniMAP-KB01
KFX-40	*Anoxybacillus* sp.	UniMAP-KB06
KFY-40	*Bacillus subtilis*	UniMAP-KB01
RFY-20	*Anoxybacillus* sp.	UniMAP-KB03
PFX-40	*Anoxybacillus* sp.	UniMAP-KB02
